# Epigenetic Modulation by Apabetalone Counters Cytokine-Driven Acute Phase Response *In Vitro*, in Mice and in Patients with Cardiovascular Disease

**DOI:** 10.1155/2020/9397109

**Published:** 2020-07-21

**Authors:** Sylwia Wasiak, Dean Gilham, Emily Daze, Laura M. Tsujikawa, Christopher Halliday, Stephanie C. Stotz, Brooke D. Rakai, Li Fu, Ravi Jahagirdar, Michael Sweeney, Jan O. Johansson, Norman C. W. Wong, Ewelina Kulikowski

**Affiliations:** ^1^Resverlogix Corp., Calgary, AB, Canada; ^2^Resverlogix Inc., San Francisco, CA, USA

## Abstract

Chronic systemic inflammation contributes to cardiovascular disease (CVD) and correlates with the abundance of acute phase response (APR) proteins in the liver and plasma. Bromodomain and extraterminal (BET) proteins are epigenetic readers that regulate inflammatory gene transcription. We show that BET inhibition by the small molecule apabetalone reduces APR gene and protein expression in human hepatocytes, mouse models, and plasma from CVD patients. Steady-state expression of serum amyloid P, plasminogen activator inhibitor 1, and ceruloplasmin, APR proteins linked to CVD risk, is reduced by apabetalone in cultured hepatocytes and in humanized mouse liver. In cytokine-stimulated hepatocytes, apabetalone reduces the expression of C-reactive protein (CRP), alpha-2-macroglobulin, and serum amyloid P. The latter two are also reduced by apabetalone in the liver of endotoxemic mice. BET knockdown *in vitro* also counters cytokine-mediated induction of the *CRP* gene. Mechanistically, apabetalone reduces the cytokine-driven increase in BRD4 BET occupancy at the *CRP* promoter, confirming that transcription of *CRP* is BET-dependent. In patients with stable coronary disease, plasma APR proteins CRP, IL-1 receptor antagonist, and fibrinogen *γ* decrease after apabetalone treatment versus placebo, resulting in a predicted downregulation of the APR pathway and cytokine targets. We conclude that CRP and components of the APR pathway are regulated by BET proteins and that apabetalone counters chronic cytokine signaling in patients.

## 1. Introduction

Liver-derived acute phase response (APR) proteins are a central component of the inflammatory response that is induced by pathogens, trauma, environmental stress, or sterile tissue injury [1]. In response to proinflammatory cytokines, hepatocytes downregulate the synthesis of several steady-state proteins (termed “negative” APR proteins) and upregulate the secretion of “positive” APR proteins [2]. IL-6 and IL-1*β* signal through transmembrane receptors on hepatocyte membrane to activate nuclear transcription factors that control APR gene transcription, including the signal transducer and activator of transcription 3 (STAT3) and the nuclear factor *κ*B (NF-*κ*B) [2]. Costimulation with IL-6 and IL-1*β* leads to coordinated loading of NF-*κ*B and STAT3 onto gene enhancers marked by increased acetylation of histone 3 lysine 27 residues (H3K27ac), resulting in a synergistic induction of gene expression [3]. Histone acetylation marks are detected by members of the bromodomain and extraterminal (BET) protein family, presenting the possibility that BETs also play a role in APR gene transcription [4]. BET proteins bind to acetylated lysines through two short peptide motifs called bromodomains (BD) 1 and 2 [4]. The BET protein BRD4 also contains a unique C-terminal domain that binds the positive transcription elongation factor P*-*TEFb, directly stimulating RNA polymerase II-dependent transcription [5, 6]. Notably, BRD4-dependent transcription is associated with the induction of transcriptional programs that drive pathogenic conditions including chronic inflammation [7, 8]. Whether BRD4 plays a direct role in regulating APR gene transcription during acute and chronic inflammation is currently unknown.

Apabetalone (RVX-208) is a small molecule inhibitor of BET proteins (BETi) that selectively targets the second BD (BD2) of BET proteins, reducing their transcriptional activity [9, 10]. Preclinical and clinical studies have shown that apabetalone modulates the expression of proinflammatory and proatherosclerotic genes in models of atherosclerosis and in patients with coronary artery or chronic kidney disease [11–17]. Apabetalone also impacts the transcription of APR genes in primary human hepatocytes (PHH), including C-reactive protein (CRP) [12, 13, 16]. Considering that CRP is a key biomarker of cardiovascular (CVD) risk assessment and treatment [18], it is important to understand its hepatic transcriptional regulation during acute and chronic inflammation. In this study, we use apabetalone *in vitro* and *in vivo* to further delineate the role of BET proteins in the transcriptional regulation of the APR pathway, with a particular focus on CRP. For the first time, we show that cytokine-mediated induction of *CRP* gene expression is BET-dependent. Further, we show that BRD4 occupancy on the *CRP* promoter increases following cytokine stimulation and that this association is inhibited by BETi. Finally, we provide evidence that apabetalone treatment of patients with cardiovascular disease (CVD) reduces the abundance of proteins within cytokine and APR pathways, potentially reducing the inflammatory risk associated with CVD.

## 2. Methods

### 2.1. Chemical Synthesis

Apabetalone and JQ1 were synthesized by NAEJA Pharmaceuticals (Edmonton, Canada) or IRIX Pharmaceuticals (Florence, SC) [12].

### 2.2. Tissue Culture

For gene expression and protein secretion studies, cryopreserved primary human hepatocytes from adult donors were plated as recommended (CellzDirect, Life Technologies). Cells were treated with compounds dissolved in DMSO (0.05-0.1%) ± cytokines (10 ng/mL) in media with 10% fetal bovine serum (FBS) (*v*/*v*) but without dexamethasone (CellzDirect, Life Technologies) for up to 72 h. For protein secretion studies, culture media were collected over the final 24 h of the experiment. HepaRG™ cells were plated as recommended (ThermoFisher Scientific). On day 6 postplating, cells were treated with cytokines ± compounds for 2 or 24 h. For short-term treatments, cells were preincubated with BET inhibitors for 1 h prior to addition of cytokines; treatments longer than 6 h received both agents simultaneously.

### 2.3. Quantification of RNA Expression

For gene expression studies, primary hepatocytes and HepaRG™ cells were harvested using the mRNA Catcher PLUS kit (ThermoFisher Scientific). Gene expression was quantified by RNA UltraSense™ One-Step qRT-PCR System (ThermoFisher Scientific). Levels of messenger RNA (mRNA) of interest were measured by human TaqMan™ Gene Expression Assays (ThermoFisher Scientific) relative to the endogenous control cyclophilin A in a duplex reaction. Data were acquired using the ViiA-7 rtPCR System (ThermoFisher Scientific).

### 2.4. ELISA

To assess the APR protein secretion, culture media from primary human hepatocyte cultures were collected over the final 24 h of treatment, flash-frozen in liquid nitrogen, and stored at -80°C prior to analysis. Secreted proteins were detected with AssayMax™ ELISA Kits (AssayPro).

### 2.5. Western Blots

Proteolysis targeting chimera (PROTAC) MZ-1 (Tocris) was added at 0.1-0.8 *μ*M for 24 h prior to cytokine treatment. HepaRG™ cell lysates were prepared in PBS+2% SDS and sonicated 3 × 10 s with a Branson SLPt sonicator (Branson Ultrasonics). Insoluble material was removed by centrifugation and protein content was assessed with the DC™ Protein Assay (Bio-Rad). Proteins were resolved by SDS-PAGE, transferred to nitrocellulose, and probed with antibodies against BRD2, BRD3, or BRD4 (Bethyl), followed by HRP*-*coupled secondary antibodies (Calbiochem). *β*-Actin was detected with antibodies coupled to HRP (Sigma-Aldrich). Luminescent signal was revealed by Amersham ECL Plus chemiluminescent reagent (GE Healthcare Life Sciences) and quantified with the Quantity One 1D analysis software (Bio-Rad).

### 2.6. Chromatin Immunoprecipitation

To assess BET protein chromatin occupancy, HepaRG™ cells were pretreated with BET inhibitors for 1 hour before addition of IL-6 and IL-1*β* (10 ng/mL) for a 2 h coincubation period. Cells were cross-linked with formaldehyde, and Active Motif Inc. (Carlsbad, CA) performed chromatin isolation and immunoprecipitation with BRD2 or BRD4 antibodies (Bethyl). Samples were processed in triplicate. Statistical significance was determined through 2-way ANOVA followed by Tukey's multiple comparison test for within-group comparison or Sidak's test for between-group comparison. A *p* value ≤ 0.05 was considered statistically significant.

### 2.7. Mouse Models

#### 2.7.1. Humanized Chimeric Mouse Model

Urokinase-type plasminogen activator (uPA)/severe combined immunodeficient (SCID) mice with livers repopulated with human hepatocytes were generated as described previously [13] (PhoenixBio, Co., Ltd., Higashihiroshima, Japan). Protocols for the animal experiments were approved by the Laboratory Animal Ethics Committee at PhoenixBio Co., Ltd. (Resolution No. 0740). *In vivo* experiments were performed in a facility approved by the Office of Laboratory Animal Welfare (OLAW). Mice received apabetalone at 150 mg/kg b.i.d. or vehicle by oral gavage for 3 consecutive days. Animals were anesthetized with isoflurane and sacrificed by cardiac puncture and exsanguination. Next, whole livers were harvested, rinsed in cold PBS, flash-frozen in liquid nitrogen, and stored at -80°C. Total RNA was extracted from chimeric livers with TRIzol® Reagent and was reverse transcribed with High-Capacity cDNA Reverse Transcription Kit, followed by rtPCR with human TaqMan™ Gene Expression Assays and TaqMan™ Gene Expression Master Mix (ThermoFisher Scientific).

#### 2.7.2. Endotoxemia Mouse Model

Prior to LPS administration, eight-week-old male C57BL/6 mice received vehicle [11] or apabetalone (150 mg/kg b.i.d., formulation EA006) by gavage for 6 days (AraVasc Inc., Moffett Field, CA). On day 7, mice received apabetalone 4 h prior to an intraperitoneal injection of *E. coli* 0111:B4 LPS (10 *μ*g per mouse) (Sigma-Aldrich) and again at the time of the LPS injection. Animals were sacrificed 24 h after the LPS injection on day 8. Liver and plasma (Li-heparin) were harvested for gene expression and protein analysis. Total RNA extracted from livers (TRIzol®) was reverse transcribed with High-Capacity cDNA Reverse Transcription Kit (ThermoFisher Scientific). Gene expression was measured as previously described [16] using mouse *β*-actin as an endogenous control. Statistical significance was determined through 1-way ANOVA followed by Tukey's multiple comparison test; *p* ≤ 0.05 was considered statistically significant.

### 2.8. SOMAscan™ Proteomic Analysis

The full design and rationale of the ASSERT study (NCT01058018) have been published previously [19]. Patients with stable coronary artery disease (CAD) and on statin therapy received placebo or 100 mg apabetalone twice per day. Baseline and end-of-study (12 weeks) plasma samples from ASSERT were analyzed using the SOMAscan™ 1.3K proteomic technology (SomaLogic Inc., Boulder, CO) that measures the relative abundance of 1305 proteins [20]. Shapiro-Wilk tests were used to determine data distribution. For normally distributed parameters, paired Student's *t-*tests were used to calculate statistical significance versus baseline, while for nonnormally distributed parameters, Wilcoxon signed-rank tests were applied versus baseline. Mann-Whitney *U* test was used to compare median percent change between apabetalone-treated patients and placebo. Proteins affected by apabetalone treatment (versus placebo) by more than 10% (*p* < 0.05) were analyzed with IPA® software using canonical pathway and upstream regulator analytics.

## 3. Results

### 3.1. Apabetalone Downregulates Basal Expression of APR Genes in Human Hepatocytes

**Figure 1:**
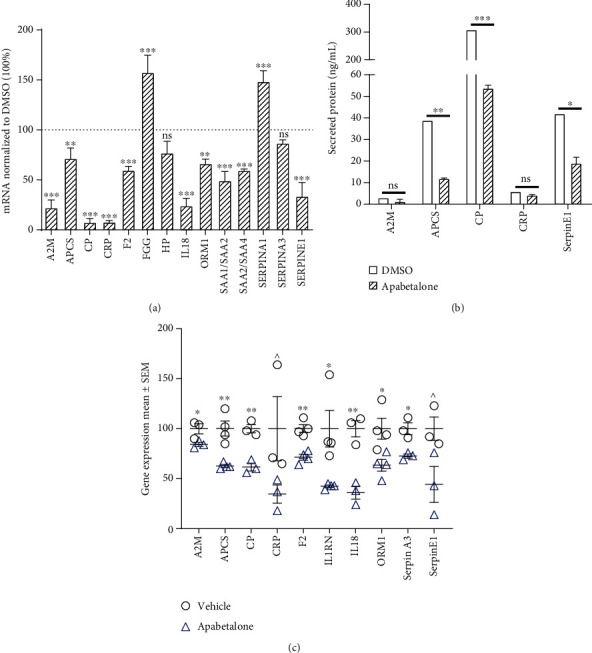
Apabetalone alters basal expression of genes within the APR pathway under noninflammatory conditions in human hepatocytes. (a) rtPCR shows that apabetalone (30 *μ*M) treatment decreases the expression of APR genes (72 h). Gene expression is normalized to vehicle (DMSO) treated cells. (b) Apabetalone (30 *μ*M) decreases APR protein secretion after 72 h of treatment. Protein secretion in the final 24 h of treatment is measured by ELISA and is expressed as nanograms per milliliter of tissue culture media. Representative data mean ± S.D. is shown. (c) APR gene expression in humanized liver of chimeric mice. Mice received 150 mg b.i.d. apabetalone or vehicle treatment for 1 to 3 days. Mean data normalized to naïve samples ± S.E.M. is shown. Statistical significance was determined through *t-*test comparison of vehicle and apabetalone treatment, where ^∗^*p* < 0.05, ^∗∗^*p* < 0.01, ^∗∗∗^*p* < 0.001, and ns means no significant difference.

Previously, we have reported that treatment with apabetalone reduced the expression of gene sets within the complement, coagulation, and APR pathways in primary human hepatocytes [12, 13, 16]. Here, we confirm the apabetalone-driven downregulation of several APR genes in nonstimulated hepatocytes obtained from human donors (72 h treatment shown in Figure 1(a)). Basal secretion of APR proteins known to correlate with CVD [21–23], including serum amyloid P (encoded by *APCS*), ceruloplasmin (encoded by *CP*), and plasminogen activator inhibitor-1 (encoded by *SERPINE1*), was also significantly lowered by apabetalone treatment (by 26-82%, Figure 1(b)). Thus, BET inhibition by apabetalone can downregulate APR gene transcription and protein abundance in noninflammatory, basal conditions *in vitro*.

To assess effects of apabetalone on basal levels of APR gene expression *in vivo*, we administered apabetalone to homozygous albumin enhancer/promoter-driven urokinase-type plasminogen activator/severe combined immunodeficient (uPA/SCID) mice with humanized liver [24]. In this chimeric liver model, replacement of mouse hepatocytes with PHH ranges from 70 to 90%, allowing for the study of human hepatocytes *in vivo*. Mice were treated with 150 mg/kg b.i.d. apabetalone or vehicle followed by liver mRNA analysis. Apabetalone reduced expression of human APR genes *A2M* (encoding *α*-2-macroglobumin), *APCS*, *CP*, *F2* (encoding thrombin), *IL1RN* (encoding IL-1 receptor antagonist), *IL18*, *ORM1* (encoding orosomucoid 1), *SERPINA1* (encoding *α*-1 antitrypsin), *SERPINE1*, and *CRP* (*p* < 0.05, except for the latter two genes where *p* ≤ 0.1) (Figure 1(c)). Overall, this data demonstrates that apabetalone can downregulate APR gene transcription in nonstimulated human primary cells and in humanized mouse liver.

### 3.2. Apabetalone Downregulates Cytokine-Induced APR Gene Expression in Hepatocytes

**Figure 2:**
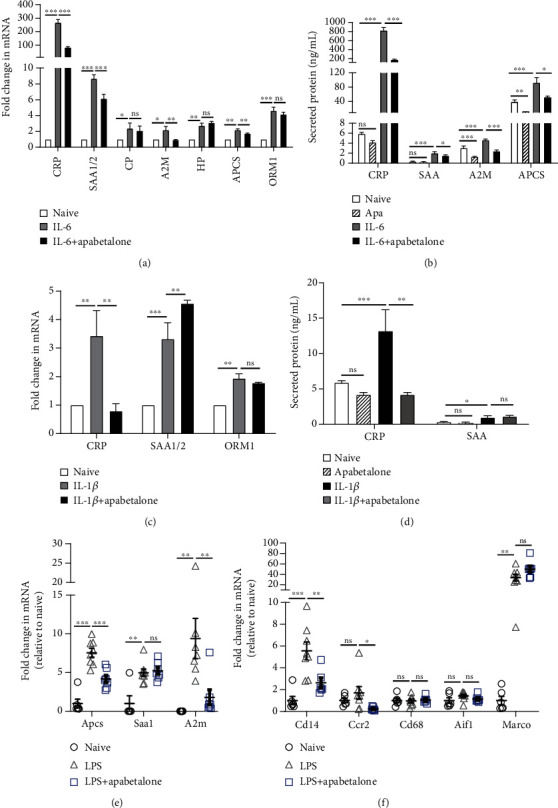
Apabetalone downregulates cytokine-induced expression of APR genes in hepatocytes. (a, b) Apabetalone (25 *μ*M) reduces IL-6 mediated induction (72 h) of *CRP*, *A2M*, and *APCS* mRNA (rtPCR) (a) and protein (ELISA) (b). Data is presented as the mean ± S.D. (c, d) Apabetalone (25 *μ*M) reduces IL-1*β*-mediated induction of *CRP* mRNA (c) and protein (d). Data is presented as the mean ± S.D. (e) Hepatic APR gene expression is downregulated by apabetalone in the LPS mouse model of systemic inflammation (rtPCR, 24 h post-LPS injection). (f) Innate immune gene expression in mouse liver (real-time PCR, 24 h post-LPS injection). (e, f) Data acquired from 6 to 8 animals per group and presented as a mean ± S.E.M. normalized to naïve mice. Statistical significance was determined through 1-way ANOVA analysis followed by Tukey's multiple comparison test, where ^∗^*p* < 0.05, ^∗∗^*p* < 0.01, ^∗∗∗^*p* < 0.001, and ns means nonsignificant.

To study hepatocyte response to BET inhibition under inflammatory conditions, we treated primary human hepatocytes with cytokines (10 ng/mL IL-6 or IL-1*β*) and apabetalone for 72 h and assessed mRNA transcription and secretion of APR proteins. *CRP*, *SAA1/2 (*encoding serum amyloid A), *CP*, *A2M*, *HP* (encoding haptoglobin), *APCS*, and *ORM1* were induced after a 72 h exposure to IL-6 (Figure 2(a)), while *CRP*, *SAA1/2*, and *ORM1* expression also increased with IL-1*β* treatment (Figure 2(c)). The *CRP* mRNA level was most sensitive to apabetalone treatment. Apabetalone reduced IL-6 mediated *CRP* induction by 69% at the mRNA level and 78% at the secreted protein level (Figures 2(a) and 2(b)) [16], whereas IL-1*β*-mediated induction of *CRP* gene and protein expression was reduced by apabetalone by 77% and 70%, respectively (Figures 2(c) and 2(d)). IL-6-induced *A2M* and *APCS* expression was less sensitive to apabetalone, with <50% reduction at the protein level (Figure 2(b)). Thus, APR gene stimulation by distinct cytokines is differentially sensitive to a BET inhibitor.

To evaluate the effect of BET inhibition on liver inflammatory responses to complex cytokine signaling *in vivo*, mice were pretreated with 150 mg/kg apabetalone (6 days) before systemic inflammation was induced by lipopolysaccharide (LPS) injection. As *CRP* gene regulation differs between mice and humans [25], expression of other model APR genes was examined in the liver post-LPS injection. *Apcs*, *Saa1*, and *A2m* genes were induced 24 h after LPS injection, and coadministration of apabetalone suppressed *Apcs* and *A2m* by 44% and 88%, respectively (Figure 2(e)). To assess the effect of apabetalone on innate immune response in the liver, we surveyed immune cell marker expression at 24 hours post-LPS injection (Figure 2(f)). Analysis revealed a suppressive effect of apabetalone on *Cd14* mRNA (encoding an LPS receptor expressed by monocytes, neutrophils, Kupffer cells, sinusoidal endothelial cells, and hepatocytes) and on *Ccr2* mRNA (encoding a chemokine receptor expressed on monocytes, Kupffer cells, and hepatic stellate cells). Apabetalone treatment did not impact the expression of macrophage and Kupffer cell markers *Cd68*, *Aif1*, and *Marco* (Figure 2(f)). Results demonstrate *in vivo* sensitivity of APR and innate immune genes to pharmacological inhibition of BET proteins during liver inflammation.

### 3.3. BET Proteins Mediate the Induction of *CRP* Gene Expression in Response to Cytokines

**Figure 3:**
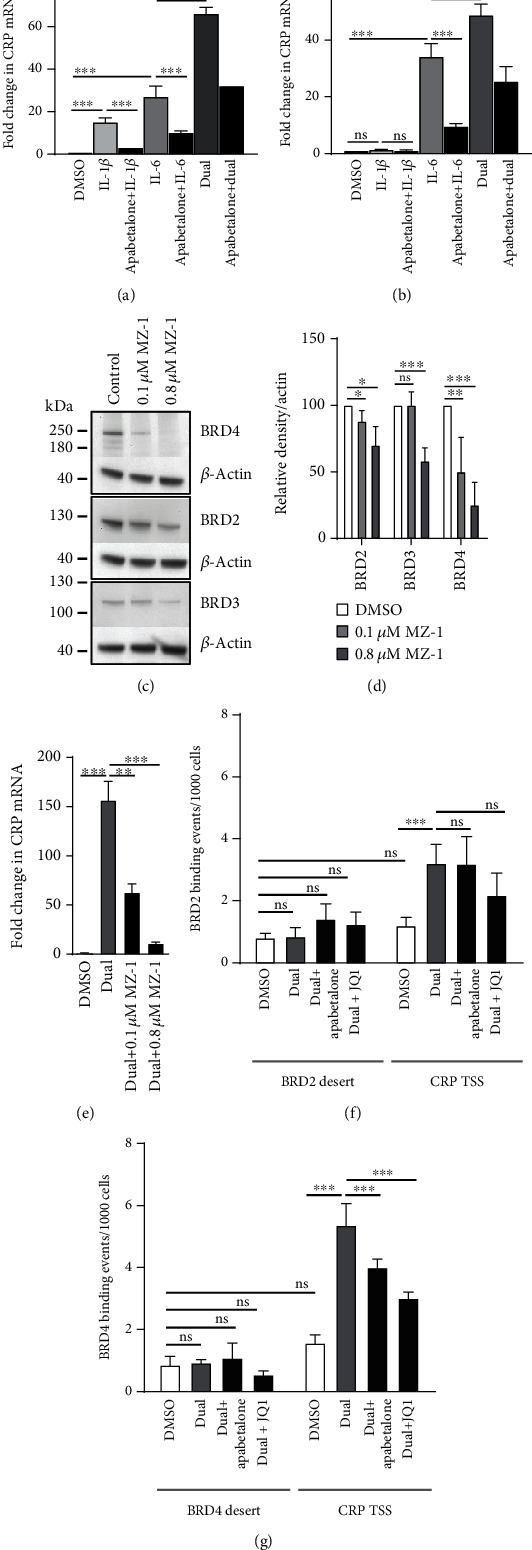
BET proteins contribute to cytokine-mediated induction of the *CRP* gene. *CRP* gene expression changes in HepaRG™ cells (a) and human primary hepatocytes (b) measured by rtPCR in response to IL-6, IL-1*β*, or combined (dual) cytokine treatment (2 h). Apabetalone pretreatment (1 h, 25 *μ*M) counters mRNA induction in response to single or dual cytokine treatment. Gene expression is graphed relative to DMSO-treated cells. Representative data from three independent repeats is shown. Data is presented as a mean ± S.D. (c) MZ-1 degrades BRD2, BRD3, and BRD4 protein in HepaRG™ cells in a dose-dependent manner. Representative Western blot data is shown. (d) Quantification of BRD2, BRD3, and BRD4 protein bands relative to *β*-actin. Data is presented as the mean of four independent replicates ± S.D. (e) BET degradation by MZ-1 significantly repressed dual cytokine-induced *CRP* transcription. Representative data from three independent repeats is shown. Data is presented as the mean ± S.D. (f, g) Dual cytokine stimulation (2 h) increases BRD2 (f) and BRD4 (g) occupancy on the *CRP* transcription start site (CRP TSS) but not in a BET protein-lacking region (desert) as determined by ChIP. Pretreatment (1 h) with apabetalone (25 *μ*M) or JQ1 (0.5 *μ*M) reduces BRD4 association with the *CRP* promoter (g). Samples were processed in triplicate. Data is presented as the mean ± S.D. Statistical significance was determined through 1-way ANOVA followed by Tukey's multiple comparison test, where ^∗^*p* < 0.05, ^∗∗^*p* < 0.01, ^∗∗∗^*p* < 0.001, and ns means no significant difference.

To examine the mechanism of *CRP* gene induction under inflammatory conditions, we used the HepaRG™ cell line, an established surrogate for human primary hepatocytes that offers robust responses to inflammatory stimuli without interdonor variability [26]. IL-6 and IL-1*β* have been previously shown to modulate *CRP* expression in hepatocytes within a few hours of stimulation [2, 3]. Here, short-term (2 h) individual cytokine treatment of HepaRG™ cells moderately increased *CRP* expression (Figure 3(a)). Simultaneous exposure to both IL-6 and IL-1*β* (dual cytokine treatment) further increased levels of the *CRP* mRNA (66-fold) (Figure 3(a)). Pretreatment with apabetalone, followed by a 2 h cotreatment with cytokines, significantly repressed dual cytokine induction of *CRP* mRNA by 52% (Figure 3(a)). *CRP* mRNA levels were similarly, albeit less robustly, modulated in primary human hepatocytes (Figure 3(b)), confirming that HepaRG™ cells are suitable for studying hepatocyte response to cytokine signaling. Preincubation of HepaRG™ cells with apabetalone did not interfere with immediate (0 to 30 minutes) signaling through IL-6 or Il-1*β* receptors as phosphorylation of STAT3 Tyr705 and NF-*κ*B p65 Ser536 was apparent within minutes of cytokine addition in the presence of apabetalone (Supplemental Figure 1). Together, this data suggests a direct involvement of BET proteins in early *CRP* gene transcription downstream of cytoplasmic inflammatory signaling.

To confirm that BET proteins play a role in cytokine-mediated *CRP* gene regulation, HepaRG™ cells were exposed to the proteolysis targeting chimeric compound (PROTAC) MZ-1 that directs BET proteins for degradation by the proteasome [27]. A 24 h incubation with either 0.1 *μ*M or 0.8 *μ*M MZ-1 decreased BRD2 and BRD4 protein abundance in cell lysates (up to 30% or 75%, respectively). BRD3 levels were not sensitive to 0.1 *μ*M MZ-1 but were reduced by 0.8 *μ*M MZ-1 (Figures 3(c) and 3(d)). 0.1 *μ*M MZ-1-driven BRD2 and BRD4 protein reduction was accompanied by a 60% decrease in dual cytokine-induced *CRP* gene expression (Figure 3(e)), indicating that these BET proteins contribute significantly to the cytokine-induced transcription of *CRP*.

To determine whether BRD2 and BRD4 proteins are directly involved in *CRP* gene transcription, we performed chromatin immunoprecipitation assays (ChIP). Dual cytokine stimulation (2 h) induced a 3.2-fold enrichment of BRD2 on the *CRP* promoter, but this increase was not sensitive to competitive BD inhibition (Figure 3(f)). Cytokine treatment also evoked a significant increase of 5.3-fold in BRD4 association with the *CRP* promoter region (CRP TSS, Figure 3(g)). This enrichment was countered by pretreatment with apabetalone (25% reduction) or JQ1 (44% reduction) indicating that it was BD-dependent (Figure 3(g)). Overall, data suggests a predominant contribution of BRD4 to the increase in *CRP* gene transcription following cytokine stimulation of hepatocytes. BRD4 inhibitors, including apabetalone, counter the BRD4 interaction with the *CRP* promoter, consistent with the decrease in *CRP* gene transcript levels measured after treatment (Figure 3(a)).

### 3.4. Apabetalone Downregulates Circulating Targets of the APR Pathway and Cytokine Signaling in CAD Patients

**Figure 4:**
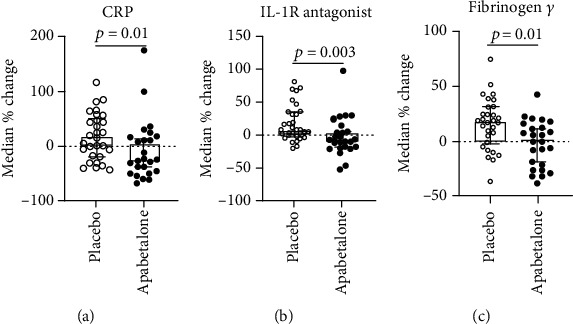
Apabetalone treatment reduces the abundance of liver-derived APR plasma proteins in CAD patients versus placebo. (a–c) Boxes represent median values of percent changes between start and end of study in each patient. Lines represent the 95% confidence interval.

Elevated circulating APR proteins are markers of chronic inflammation and correlate with CVD [28]. To assess the effects of BET inhibition by apabetalone on circulating inflammatory mediators, SOMAscan™ 1.3K proteomic analysis [20] was performed on plasma samples from patients with stable CAD on standard-of-care therapy, treated with placebo or 100 mg b.i.d. apabetalone for 12 weeks (*n* = 55; as described in [19]). Changes in plasma protein abundance specific to apabetalone treatment (>10% difference versus placebo, *p* < 0.05) were analyzed using the “canonical pathway” and “upstream regulator” bioinformatics tools with the Ingenuity® Pathway Analysis (IPA®) software. The “canonical pathway” analytic tool ranked “APR signaling” as the top pathway significantly downregulated by apabetalone in plasma from CAD patients (versus placebo, *p* value = 2.4 × 10^−10^, *z* − score = −2.1) (Table 1). The IPA® “upstream regulator” analysis was used to group plasma proteins affected by apabetalone treatment as transcriptional targets of specific upstream activators. This analysis highlighted downstream signaling by LPS, IL-6, interferon *γ* (IFN*γ*), IL-1*α*, the IL-6-like cytokine oncostatin M (OSM), and the nuclear factor *κ*B subunit 1 (NFKB1), as significantly suppressed by apabetalone versus placebo (predicted downregulation with a *z* − score < −2, *p* value < 0.05) (Table 1). No significant changes were detected in plasma levels of IL-6, interferon *γ* (IFN*γ*), and IL-1*β* between placebo- and apabetalone-treated CAD patients (not shown). However, as indicated by the IPA® “upstream regulator” analysis, a number of circulating plasma proteins regulated by these cytokines, including CRP, IL-1R antagonist, and fibrinogen *γ*, were reduced by apabetalone treatment (Figure 4). These are liver-derived APR proteins, indicating that apabetalone impacts APR protein secretion from hepatocytes in humans. Taken together, the plasma proteomics data from CAD patients demonstrates the modulatory effect of apabetalone on circulating APR proteins and inflammatory pathways linked to progression of CVD.

## 4. Discussion

Systemic inflammatory signaling that accompanies chronic disease leads to altered production of APR proteins by the liver [1]. Changes in the transcriptome and the proteome associated with cytokine signaling in hepatocytes have been extensively investigated, but mechanisms that control APR gene transcription are not fully understood. This is due to the complexity of hepatocyte responses to cytokines, glucocorticoids, and growth factors secreted during APR that lead to activation of multiple parallel pathways [2, 29]. Transcription factors previously implicated in the regulation of APR expression differ substantially between genes and show dynamic time- and stimulus-dependent responses [2, 29]. Members of the STAT, NF-*κ*B, C/EBP, hepatocyte nuclear factor, AP-1, and Smad protein families have been detected on APR gene promoters, including *CRP*, in hepatocarcinoma cells and in primary hepatocytes [2, 29–32] (and references therein). Here, we identify BET proteins as epigenetic readers that regulate APR gene transcription. We use the BET inhibitor apabetalone, developed to treat CVD, as a tool compound to counter the activity of BET proteins in noninflammatory and inflammatory conditions in hepatocytes *in vitro*, in mice and in CAD patients.

The best characterized BET protein, BRD4, plays a crucial role in inflammatory responses [7, 8]. BRD4 occupies transcriptionally active chromatin regions through its interactions with H3K27ac [29, 30, 33]. In mouse hepatocytes, IL-1*β* and IL-6 dual cytokine signaling has been shown to increase the abundance of H3K27ac, STAT3, NF-*κ*B, and RNA polymerase II in the vicinity of induced APR genes [3]. BRD4 cooperates with both STAT3 and NF-*κ*B to transactivate RNAPII in nonhepatocytes [31, 32, 34]; thus, it is conceivable that it plays a similar role in activated hepatocytes. Here, we provide evidence that BRD4 is a novel transcriptional regulator required for *CRP* expression in stimulated hepatocytes. Degradation of BET proteins by the proteolysis-inducing compound MZ-1 potently suppresses *CRP* gene induction by IL-6 and IL-1*β*, reinforcing the notion that BET proteins are required for stimulation-dependent transcription. This is likely a direct effect, as BRD2 and BRD4 recruitment to the *CRP* promoter is detected early on during dual cytokine stimulation (2 h), and a short-term treatment with apabetalone or JQ1 (a pan-BETi with a different scaffold) counters the *CRP* promoter occupancy by BRD4 and the C*RP* gene induction by cytokines. Further investigation will determine whether BRD2 and BRD4 cooperate with other transcription factors to promote hepatic *CRP* transcription during inflammation.

We also show that long-term BET inhibition with apabetalone treatment (up to 72 h) downregulates basal levels of several APR genes in human hepatocytes. This coordinated sensitivity of APR gene expression to apabetalone indicates that BET proteins are involved (directly or indirectly) in regulating this key hepatic function. Of note, suppression of APR genes by apabetalone was consistent across hepatocyte models, including *in vitro*-cultured human primary cells and chimeric mice with humanized livers. Moreover, mRNA and protein expression of APR proteins linked to CVD, including serum amyloid P, ceruloplasmin, and plasminogen activator inhibitor-1 [21–23], was also lowered by apabetalone treatment. Although serum amyloid P and plasminogen activator inhibitor-1 were not reduced in stable CAD patients analyzed here, their downregulation by apabetalone was previously observed in patients with advanced CAD [16] and in chronic kidney disease patients [17], respectively. Thus, disease type and severity may influence the sensitivity of the APR proteins to apabetalone.

During endotoxemia, apabetalone suppressed hepatocyte response to LPS, as shown by downregulation of mouse model APR genes *APCS* and *A2M*. This *in vivo* effect likely results from direct modulation of gene transcription by apabetalone as demonstrated in *in vitro* experiments with stimulated hepatocytes. However, apabetalone may also act indirectly by impacting infiltrating or liver-resident innate immune cells, which stimulate hepatocytes to produce APR proteins during endotoxemia. Innate immune marker profiling showed that apabetalone suppressed the inflammatory expression of the *Cd14* gene, which encodes an LPS coreceptor displayed on monocytes, neutrophils, Kupffer cells, sinusoidal endothelial cells, and hepatocytes [35, 36]. As previously reported [37], *Cd14* expression level is relatively low in normal liver but sharply increases following LPS treatment (also shown in Figure 2(f)), facilitating LPS uptake by cells. Downregulation of *Cd14* mRNA by BETi treatment would potentially reduce LPS signaling in the liver, attenuating the hepatocyte inflammatory response [38, 39]. Apabetalone also reduced the mRNA level of *Ccr2*, a key chemokine receptor mainly found on the surface of infiltrating monocytes, macrophages, and resident Kupffer cells [40]. *Ccr2* functions to recruit these cells to the site of inflammation, and its reduction by apabetalone may attenuate immune cell activity in the liver. Benefits of BETi treatment for inflammatory liver disease are currently being investigated [41, 42].

Enhanced inflammatory signaling accompanies several chronic human diseases including CVD, where elevated plasma APR proteins are being used as biomarkers of systemic inflammation [35]. Stable CAD patients studied here had elevated hsCRP levels (3.9 ± 0.3 mg/L) despite standard-of-care therapy [16], reflective of elevated systemic cytokine signaling. To study the effect of apabetalone in CAD patients, we examined changes in 1300+ plasma proteins in response to drug treatment over the course of 3 months, versus placebo. Bioinformatics analysis of the plasma proteome pre- and posttreatment showed that apabetalone significantly modulates levels of multiple components of the canonical “APR signaling pathway,” resulting in a predicted inhibition of the APR pathway in treated patients (Table 1). APR proteins most significantly downregulated by apabetalone were the IL-1R antagonist (-13.8%, *p* = 0.003), fibrinogen *γ* chain (-16.9%, *p* = 0.01), and CRP (-42.3%, *p* = 0.01) (Figure 4), each of which positively correlates with an increased risk of CVD [18, 36]. Apabetalone-mediated reductions in these liver-derived plasma APR proteins support observations from both cellular and animal studies (this report and [12]). Further, bioinformatics analysis of the plasma proteome showed that circulating APR proteins affected by apabetalone are transcriptional targets of inflammatory mediators IL-6, IFN*γ*, IL-1*α*, oncostatin M (OSM), LPS, and NF-*κ*B (Table 1). Consistent with this finding, several other cytokine targets were also downregulated by apabetalone in patients' plasma (Table 1) including stromelysin-2/MMP10 (-29%, *p* < 0.008), RANTES/CCL5 (-29%, *p* < 0.04), TWEAK/TNFSF12 (-21%, *p* < 0.002), osteopontin/SPP1 (-16%, *p* < 0.03), epiregulin/EREG (-13%, *p* < 0.01), PARC/CCL18 (-13%, *p* < 0.03), and LIGHT/TNFSF14 (-11%, *p* < 0.004). These proteins are linked to atherosclerotic plaque development and rupture, and their circulating levels correlate with CVD risk [18, 36–42]. Thus, downregulation of cytokine pathways that lead to APR protein expression may reduce CVD risk in apabetalone-treated patients [43, 44].

## 5. Conclusions

BET inhibition by apabetalone counters cytokine signaling in hepatocytes, leading to reduced APR gene expression and protein secretion, including *CRP*. Reduction of the *CRP* gene expression by apabetalone during early cytokine signaling is at least partly mediated by BRD4 removal from the *CRP* promoter. Apabetalone treatment downregulates circulating cytokine targets, including the APR pathway components, in stable CAD patients on standard-of-care therapy, potentially dampening chronic inflammatory signaling in a disease. Apabetalone's impact on inflammatory mediators could contribute to a reduction in CVD risk and disease outcomes [43, 44].

## Figures and Tables

**Table 1:** Apabetalone downregulates transcriptional targets of inflammatory mediators in the CAD patient plasma as predicted by IPA® “canonical pathway” and “upstream regulators” analysis.

Ingenuity® Pathway Analysis	Pathway/regulator^a^	Activation *z*-score^b^	*p* value of overlap^c^	Target molecules in dataset (gene symbols)^d^
Canonical pathway	APR signaling	-2.1	2.4 × 10^−10^	C4A/C4B, C3, IL1RN, CRP, MAPK9, FGA/FGB/FGG, AGT
Upstream regulators	LPS	-3.1	8.9 × 10^−13^	ADIPOQ, AGT, BCL6, C3, CCL18, CCL5, CDH1, CDH5, CRP, CSF1R, EREG, FGA/FGB/FGG, IL1RN, IL2, KIT, MAPK9, MB, MMP10, NCR3, POMC, SPP1, TIMP2, TNFSF14, VTN
Upstream regulators	IL-6	-2.6	7.1 × 10^−15^	AGT, BCL6, C3, CCL5, CDH1, CDKN1B, CRP, EREG, FGA/FGB/FGG, GCG, IGFBP6, IL1RN, IL2, KIT, MMP10, POMC, SPP1
Upstream regulators	IFNG	-2.6	2.5 × 10^−10^	ADIPOQ, AGT, C3, C4A/C4B, CCL18, CCL5, CDH1, CDKN1B, CSF1R, CTSD, ERAP1, FGA/FGB/FGG, IL1RN, IL2, MMP10, NCR3, POMC, SPP1, TNFSF12
Upstream regulators	OSM	-2.0	6.5 × 10^−7^	CCL5, SPP1, MMP10, IGFBP6, FGA/FGB/FGG, CDKN1B, CDH1, CRP, POMC
Upstream regulators	IL1A	-2.0	2.0 × 10^−6^	CCL5, IL1RN, IL2, KIT, MMP10, POMC, SPP1
Upstream regulators	NFKB1	-1.8	5.2 × 10^−5^	CCL5, CRP, FGA/FGB/FGG, IL1RN, IL2, MMP10

Plasma proteins affected by apabetalone treatment by more than 10% (versus placebo, *p* < 0.05) were analyzed with the IPA® “canonical pathway” and “upstream regulator” analytics. ^a^APR: acute phase response; LPS: lipopolysaccharide; IL-6: interleukin 6; IFNG: interferon *γ*; OSM: oncostatin M; IL1A: interleukin 1*α*; NFKB1: nuclear factor *κ*B subunit 1. ^b^IPA® z-score compares the observed differential regulation of a gene in the dataset to changes predicted by the literature which can be either “activating” or “inhibiting.” *z* − score < −2 predicts downregulation within a gene set associated with a transcriptional regulator. ^c^The overlap *p* value measures whether there is a statistically significant overlap between the dataset genes and the genes that are regulated by a transcriptional regulator. It is calculated using Fisher's exact test, and significance is attributed to *p* values < 0.01. ^d^Apabetalone target proteins that contribute to IPA**® “**canonical pathways” or “upstream regulators” are listed as gene symbols.

## Data Availability

The datasets generated during and/or analyzed during the current study are available in this manuscript.

## Abbreviations

A2M: *α*-2-Macroglobulin
AP*-*1: Activator protein 1
APCS: Amyloid P component, serum
APR: Acute phase response
BD: Bromodomain
BET: Bromodomain and extraterminal
BETi: BET inhibitor
BRD: Bromodomain and extraterminal
CAD: Coronary artery disease
C/EBP: CCAAT/enhancer binding protein
ChIP: Chromatin immunoprecipitation
CKD: Chronic kidney disease
CP: Ceruloplasmin
CRP: C-reactive protein
CVD: Cardiovascular disease
H3K27: Histone 3 lysine 27
HNF: Hepatocyte nuclear factor
HP: Haptoglobin
HsCRP: High-sensitivity CRP
IL: Interleukin
IL-1RA: Interleukin 1 receptor antagonist
IPA®: Ingenuity® Pathway Analysis
LPS: Lipopolysaccharide
MACE: Major acute cardiac event
NF-*κ*B: Nuclear factor *κ*B
ORM1: Orosomucoid 1
OSM: Oncostatin M
PCR: Polymerase chain reaction
PHH: Primary human hepatocytes
P-TEFb: Positive transcription elongation factor b
PROTAC: Proteolysis targeting chimera
RNAPII: RNA polymerase II
rtPCR: Real-time PCR
SAA: Serum amyloid A
SAP: Serum amyloid P
STAT3: Signal transducer and activator of transcription 3
TNF: Tumor necrosis factor.

## Supplementary Materials

Supplementary Materials Supplemental Figure 1: apabetalone does not affect early signaling steps by IL-6 and IL-1*β*.
