# Supplementary material for: Epigenetic Modulation by Apabetalone Counters Cytokine-Driven Acute Phase Response In Vitro, in Mice and in Patients with Cardiovascular Disease
Source: Cardiovasc Ther. 2020 Jul 21;2020:9397109. doi: 10.1155/2020/9397109 (PMC7416228; doi:10.1155/2020/9397109)
Supplement: Supplementary Materials — Supplemental Figure 1: apabetalone does not affect early signaling steps by IL-6 and IL-1β. [file 9397109.f1.pdf]

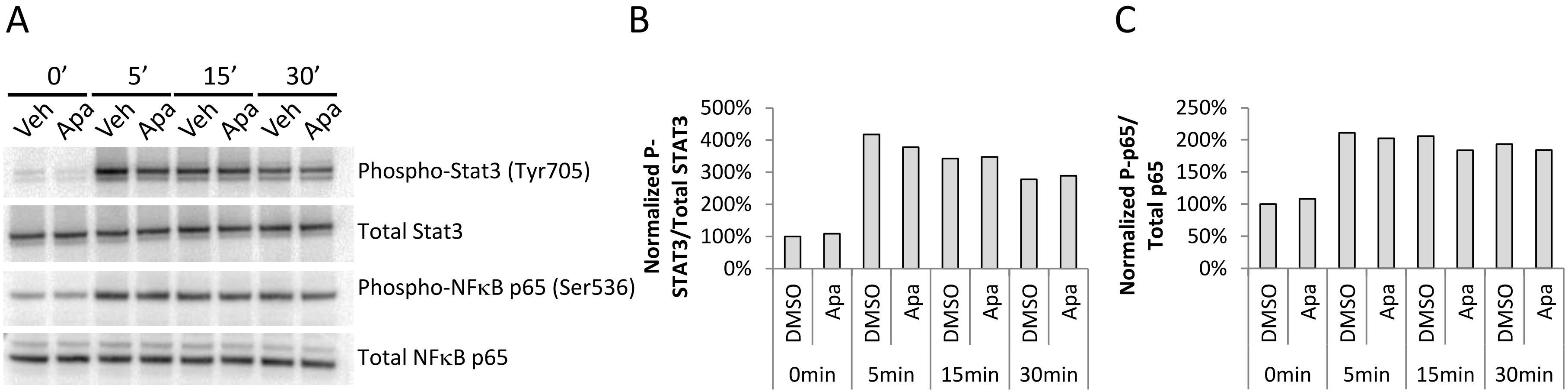

Supplemental Figure 1 **Apabetalone does not affect early signaling steps by IL-6 and IL-1β.** A) HepaRG™ cells were pre-incubated with 0.1% DMSO or 25μM apabetalone for 1 hour, followed by a co-stimulation with 10 ng/mL IL-6 and 10 ng/mL IL1β for 5, 15 or 30 minutes. Equal amounts of total cell lysate were resolved by SDS-PAGE, transferred to nitrocellulose and probed with antibodies against total STAT3 (Cell Signaling Technology #9139), phospho-STAT3 (Cell Signaling Technology #9145), total p65 (Abcam #16502) and phospho-p65 (Cell Signaling Technology #3033), followed by HRP-coupled secondary antibodies (Calbiochem). B) and C) Luminescent signal was quantified with the QuantityOne 1D analysis software (BioRad). Phosphorylated protein signal was normalized to total amount of non-phosphorylated protein present in each lane and to DMSO baseline (0’).
